# Sensitivity of PCR Assays for Murine Gammaretroviruses and Mouse Contamination in Human Blood Samples

**DOI:** 10.1371/journal.pone.0037482

**Published:** 2012-05-21

**Authors:** Li Ling Lee, Lin Lin, David S. Bell, Susan Levine, Maureen R. Hanson

**Affiliations:** 1 Department of Molecular Biology and Genetics, Cornell University, Ithaca, New York, United States of America; 2 Department of Pediatrics, State University of New York, Buffalo, New York, United States of America; 3 Private Practice, New York, New York, United States of America; National Institute of Health, United States of America

## Abstract

Gammaretroviruses related to murine leukemia virus (MLV) have variously been reported to be present or absent in blood from chronic fatigue syndrome/myalgic encephalomyelitis (CFS/ME) patients and healthy controls. Using subjects from New York State, we have investigated by PCR methods whether MLV-related sequences can be identified in nucleic acids isolated from whole blood or from peripheral blood mononuclear cells (PBMCs) or following PBMC culture. We have also passaged the prostate cancer cell line LNCaP following incubation with plasma from patients and controls and assayed nucleic acids for viral sequences. We have used 15 sets of primers that can effectively amplify conserved regions of murine endogenous and exogenous retrovirus sequences. We demonstrate that our PCR assays for MLV-related *gag* sequences and for mouse DNA contamination are extremely sensitive. While we have identified MLV-like *gag* sequences following PCR on human DNA preparations, we are unable to conclude that these sequences originated in the blood samples.

## Introduction

The 2009 report [1] that XMRV (xenotropic murine leukemia virus-related virus) was associated with CFS sparked our interest in examining additional populations to determine whether we could replicate the results and observe variability from the originally reported sequences. CFS/ME is a debilitating illness without a known cause and no generally effective treatment [2]–[6]. Aspects of the illness, including a number of outbreaks [7], [8], are consistent with involvement of a virus. Our initial attempts to utilize published primers in PCR assays to detect XMRV failed; however, some experiments resulted in identification of *gag* sequences similar to MLV. With the report in 2010 [9] of detection of MLV-like *gag* sequences in CFS patient blood samples, we decided to explore our findings further. As reports of laboratory [10], [11] and reagent contamination [12] began to appear, we investigated the possibility of spurious results and the possible sources of the sequences we observed.

Here we describe our analysis of samples from patient and control populations from rural and urban New York. We have measured the sensitivity of nested and single-round PCR assays for MLV-like *gag* sequences in human whole blood and PBMCs, and in mouse DNA. We have also identified additional sets of primers that can be used to search for the presence of other conserved regions of murine gammaretroviruses. We have performed spiking experiments to determine the sensitivity of mitochondrial DNA (mtDNA) and IAP assays for mouse cellular DNA contamination.

We are unable to identify the provenance of the MLV-like *gag* sequences we have detected. Therefore, we cannot conclude that MLV-related *gag* sequences are present in the blood samples in this study. We demonstrate that our PCR assays are highly sensitive and specific for MLV-related viruses. Whether a retrovirus is involved in inciting or maintaining CFS/ME will require further investigation using other types of assays.

## Materials and Methods

### Ethics statement and study subjects

Patients with CFS fulfilled Fukuda criteria [13] and were identified by two physicians experienced with CFS/ME. All patients gave written informed consent for the use of their blood samples for research concerning CFS/ME and the study was approved by Institutional Review Board at Cornell University, Ithaca, NY (approval # 1005001407). One group of participants was recruited by David Bell, M.D., Lyndonville, New York, where an outbreak occurred in 1984–1986. The cohort contained 10 individuals who are severely ill with CFS, 10 individuals who fulfilled Fukuda criteria at one time but now consider themselves recovered, and 20 individuals who have never been diagnosed with CFS (controls). Not all study subjects recruited by David Bell reside in Lyndonville and only some were part of the outbreak population. Susan Levine, M.D., provided samples from 20 CFS patients and 4 healthy controls visiting her practice in Manhattan, New York. 12 controls who have never been diagnosed with CFS were recruited from Ithaca, New York. Health status of subjects was unknown to the individuals who performed experiments with blood samples.

### Collection and processing of blood samples

Blood samples were collected in vacutainers by phlebotomists and sent to us via overnight courier or hand-carried to the laboratory. Ithaca samples were maintained at room temperature for 20 h to mimic the shipment by courier. Blood samples were processed in a sterile containment hood within 24 h of draw. All samples were handled under aseptic conditions. EDTA blood collection tubes were used in all three locations.

1 ml of unprocessed whole blood from some samples was stored at −80°C. Following centrifugation of whole blood at 500 *g* for 5 min, plasma samples were stored in 1 ml aliquots at −80°C. The blood cells were fractionated by Ficoll-Paque (GE Healthcare, Piscataway, NJ) gradient and the PBMC buffy coat was washed with PBS. The resultant PBMC pellet was washed with PBS again and divided into 3 fractions of which one was cultured, one resuspended in 1 ml TRIzol (Invitrogen, Carlsbad, CA) for RNA preparation, and one resuspended in 700 µl CTAB for DNA preparation.

The containment hood used for this study was in a separate room from the main laboratory, with a designated set of pipettes and centrifuges. All disposable laboratory consumables were sterile and purchased from Corning (Corning, NY).

### PBMC culture

PBMCs were cultured in 25 cm^2^ flasks in 10 ml complete medium [RPMI medium 1640 with L-glutamine, 10% fetal bovine serum (FBS), liquid penicillin-streptomycin (all from Invitrogen), 5 µg/ml ciprofloxacin (Sigma-Aldrich, St. Louis, MO)], supplemented with 1 µg/ml phytohemagglutinin (Sigma-Aldrich) and 2.2 µg/ml interleukin-2 (ZeptoMetrix, Buffalo, NY), and maintained at 37°C and 5% carbon dioxide (CO_2_). PBMCs were collected at 3–5 d with media replenishment, and 8–10 d from initial culturing.

### LNCaP culture

The LNCaP FGC cell line was from American Type Culture Collection (ATCC, Manassas, VA**)** and maintained at 37°C and 5% CO_2_, on complete medium and subcultivated using TrypLE Select (Invitrogen). Co-incubation of LNCaP cells with plasma samples was conducted to determine viral transmission. In sterile 15 ml conical tubes, LNCaP cells (1 of 16 parts from 80% confluent 75 cm^2^ flask) in serum-free RPMI medium 1640 with 150 µl plasma were incubated in a 37°C water-bath for 1 h, followed by centrifugation at 500 g for 5 min. The supernatant was removed and cells were resuspended in complete medium and cultured in 25 cm^2^ flasks with 10 ml of media. Subcultivation of LNCaP cells that were exposed to plasma (‘LNCaP-plasma cells’) were performed when attached cells were grown to 70–80% confluence, usually at 1 4 ratio every 4 to 5 d. The remainder of the LNCaP-plasma cells were divided into 3 tubes and stored as pellets at −80°C. LNCaP-plasma cells were maintained through 6 passages and then stored frozen. Tissue culture of LNCaP stock cell line was always performed separately and/or prior to tissue culture of LNCaP-plasma cells.

### Nucleic acid preparations

Nucleic acids were prepared in a designated ‘nucleic acids' UV workstation with a designated set of pipettes, in a room separate from blood processing, tissue culture, and analysis of PCR products. The space was exposed to UV light for at least 10 min before use and all tubes were irradiated in an UV cross-linker (Stratagene, Santa Clara, CA). Manufacturer-sterilized filter tips were used in all experiments.

RNA samples were prepared using TRIzol and cDNA samples were synthesized from 750 ng RNA per SuperScript VILO cDNA synthesis kit reaction. These reagents were from Invitrogen and experiments were carried out according to manufacturer's guidelines.

DNA was prepared from whole blood samples with the QIAamp DNA blood mini kit (Qiagen, Valencia, CA). In order to isolate DNA from PBMCs or cultured cells, 1–3 million cells were resuspended in 700 µl of 2× CTAB and incubated at 65°C for 30 min. At room temperature, 700 µl of chloroform was added and the sample mixed by vortex. Following centrifugation at 4°C for 10 min at 12000 rpm, the upper aqueous layer was collected in a fresh 1.5 ml tube and chloroform-extracted again. An equal volume of isopranol was added to the aqueous layer to precipitate the DNA, which was washed twice with 70% ethanol and air-dried. The DNA pellet was resuspended in 100 µl water and stored frozen.

### PCR amplification

PCR mixes were set up in a designated ‘PCR’ UV workstation with a designated set of pipettes, in a room separate from blood work, tissue culture, and analysis of PCR products. The space was exposed to UV light for at least 10 min before use and all tubes were irradiated in an UV cross-linker. Manufacturer-sterilized filter tips were used in all experiments.

Routine PCR was with a Bio-Rad C1000 thermal cycler and consisted of denaturation at 92°C for 2 min; cycles of 92°C for 30 sec, annealing for 30 sec, extension at 72°C for 1 min per kb product; and final extension for 10 min. PCR reactions were 50 µl total with Hotstart-IT FideliTaq master mix (USB), 2.5 mM MgCl_2_, 400 nM of each primer, and either 500 ng genomic DNA (gDNA) or 5 µl of cDNA. 5 µl of the first PCR product were added as template for the nested PCR.

New PCR primers were designed (manufactured by IDT, Coralville, Iowa) and PCR conditions were optimized as necessary. Optimization of PCR conditions were carried out with additional 20 nM of each VAMP2 primer in each reaction as positive control for successful PCR. All experiments with mouse DNA were performed in a different laboratory. All PCR primers and amplification conditions used are in Tables S1 and S2. Mouse tail DNA from genotype C57BL/6J (gift from Ling Qi's laboratory, Cornell) was used to determine the limits of detection. An amplicon from a human blood sample was produced with primers 419F/1154R [1] and cloned into vector pCR2.1 (Invitrogen) for use in spiking experiments.

### Virus culture

40,000 DERSE cells were cultured in 25 cm^2^ flasks with 10 ml RPMI medium 1640 containing 10% FBS and 1 µg/ml puromycin, for 72 h at 37°C and 5% CO_2_ (all reagents were from Invitrogen). DERSE cells were then incubated with 250 µl plasma in 2 ml of fresh RPMI medium (no serum) for 2 h at 37°C, 5% CO_2_. Cultures were observed to ensure cell attachment and an additional 3 ml RPMI medium with penicillin-streptomycin was added to each flask and incubated overnight. The following day, the media were replaced with 10 ml RPMI medium containing 10% FBS and penicillin-streptomycin and incubated for 3 d. Cultures were subcultivated 1 4 using TrypLE Select every 3–4 d. Cultures were monitored for green fluorescent protein (GFP) fluorescence and to prevent growth to confluence. A total of 32 cultures were treated the same way with the same media and maintained: 30 of DERSE cells that were co-incubated with plasma, and two of DERSE cells only.

As a positive control, two separate flasks of DERSE cells were incubated with supernatant from the 22Rv1 cell line, which contains XMRV (gift from Vineet Kewalramani, NCI, Frederick) and monitored for GFP fluorescence. This experiment was conducted in a colleague's laboratory, using their equipment, by a researcher who did not handle any of the other experiments in this study.

### PCR and DNA sequencing

Following PCR, samples were separated by 1 or 2% agarose gel electrophoresis with ethidium bromide staining. Any amplicons of the expected size were purified from agarose gels using the PureLink kit (Invitrogen) and sequenced at the Cornell University Life Sciences Core Laboratories Center.

### Nomenclature and Genbank Accession Numbers

In order to label the *gag* sequences we obtained, we have devised the following code to indicate which sample was used in the PCR reactions from which *gag* fragments were obtained. 1: severe CFS, 2: recovered CFS, 3: non-CFS control, A: whole blood gDNA, B: fresh PBMC cDNA, C: Fresh PBMC gDNA, D: 3–5 d cultured PBMC gDNA, E: 8–10 d cultured PBMC gDNA, F: LNCaP incubated with plasma gDNA. LN1: initial batch of cultured LNCaP cells. LN2: second batch of cultured LNCaP cells. P4: passage 4 LNCaP cells, P6: passage 6 LNCaP cells. For example 5A1 refers to gDNA of whole blood of subject 5, who has severe CFS. Genbank accession numbers are JQ684649, JQ684650, JQ684651, JQ684652, JQ684653, JQ684654, JQ684655, and JQ684656.

## Results

### MLV-like *gag* sequences were obtained following PCR of gDNA and cDNA from PBMCs from subjects in Western New York

Blood samples were obtained from subjects in New York State living in the vicinity of Lyndonville, where a CFS outbreak occurred in the mid-1980's [14]. PBMCs were isolated and examined directly or cultured in order to determine whether MLV-related viruses could be detected. Plasma was incubated with LNCaP cells to determine whether infectious virus was present that could infect a human cell line. PCR was performed with gDNA and cDNA produced from uncultured and 5-day and 9/10-day cultured PBMCs and plasma-inoculated LNCaP cells. In addition to the nested gagO/gagI primers utilized by Lombardi et al. [1] and Lo et al. [9], we designed another set of primers for gagL PCR (from the *gag* leader region) (Table S1) for use in single-round, 45-cycle PCR in order to minimize the possibility of environmental contamination with mouse sequences that might occur during the manipulations needed for nested PCR. Conditions for this new set of primers were optimized by performing a gradient of 8 annealing temperatures from 52°C to 59°C. The optimization was performed by using a sample that had resulted in amplification with the gagO/gagI nested PCR. A product was observed with annealing temperature of 54.8°C and this temperature was used for gagL PCR (Table S2). As a positive control for successful PCR during optimization, 20 nM of VAMP2 primers were added to each reaction. Amplicons corresponding to the size expected for VAMP sequences were observed with PCR annealing temperatures ranging from 50°C to 59°C.

PCR products corresponding to *gag* sequences were obtained from whole blood or PBMC DNA from 6 different samples with single-round gagL PCR. When nested gagO/gagI PCR was performed, 4 samples resulted in fragments corresponding to *gag* sequences. Taken together, 5 samples from severe patients, 2 from recovered CFS patients, and 3 from control subjects resulted in detection of gag PCR products. Samples from one severely ill and one recovered patient exhibited PCR products with both gagL and gagO/gagI amplifications. All of these samples were negative in mouse mtDNA assays. All PCR results were confirmed by sequencing.

### No XMRV observed by PCR or DERSE cell co-culture

Detectors of Exogenous Retroviral Sequence Elements (DERSE) indicator cells [15] were used for detection of XMRV-related viruses in plasma of 10 of each health status types from Bell's cohort. The DERSE cell lines are LNCaP cells carrying a MLV-based vector which will result in expression of GFP if MLV-related gammaretroviruses capable of infecting human cell lines, such as XMRV or X-MLVs, are present.

No *gag* sequences with the XMRV-characteristic deletion relative to MLV in the *gag* leader region were ever observed in any PCR assays on any type of samples. Furthermore, co-incubation of DERSE cells with plasma of 20 patient and 10 control samples from Western New York produced no GFP-expressing cells, indicating absence of detectable infectious XMRV. Incubation of the DERSE cells with XMRV virions from 22Rv1 was performed as a positive control, and resulted in the rapid detection of GFP-expressing cells (Fig. S1).

### MLV-like *gag* sequences detected in LNCaP cultures

The gagL and gagO/gagI PCRs were carried out on gDNA isolated from LNCaP cells at passage 4 or passage 6 following incubation with plasma from 40 subjects. For every 10 plasma-incubated cultures, two uninoculated LNCaP cultures were grown alongside with the same media and conditions to serve as negative controls. All the samples were negative for mouse *cox2* DNA according to nested PCR with coxO-coxI primers. *gag* sequences were detected in gDNA preparations following both the gagO/gagI and gagL PCR. Two samples gave gagO/gagI PCR products: 11F2-P4 and 14F2-P4. With gagL PCR, 7 samples were positive: 2F1-P6, 4F1-P6, 9F1-P6, 10F3-P6, 11F2-P4, 12F2-P6, and 13F3-P6. In addition, 5 additional samples irreproducibly gave gagO/gagI PCR products and 11 sometimes were PCR positive for gagL; due to the inconsistency, we decided to score these as negative. For each sample, at least 3 PCR reactions were performed on 3 different days. Sometimes *gag* fragments were not detected in the same sample in which they had previously been found; also, sometimes *gag* sequences were found at passage 4 but not at passage 6, as well as vice versa. None of the uninoculated LNCaP cultures gave *gag* PCR products with either set of primers when a small number of PCR reactions were run contemporaneously with the plasma-incubated cultured cells.

In order to determine whether low-level contamination might be occurring, we tested the uninoculated LNCaP master cell line by gagL PCR on a larger scale of replicates. On the same day, one sample of LNCaP gDNA designated LN1 was tested with gagL PCR using HotStart-IT FideliTaq master mix (USB) and OneTaq® 2× master mix (NEB) separately, each with 42 LNCaP gDNA aliquots and 6 no-template negative controls. gagL sequences were confirmed in 4/42 of each of the PCR. None of the negative control samples were positive. LN1 showed *gag* sequences in 9.5% (8/84) PCR tests. 10 replicates of LN1 IAP PCR conducted with HotStart-IT FideliTaq Master Mix (USB) were all negative. Furthermore, 24 replicates of USB and 14 of NEB master mixes were tested for IAP PCR to rule out contamination in the reagents and all 38 were negative.

Because LN1 appeared to contain *gag* sequences, an earlier sample of the LNCaP master cell line gDNA, named LN2, was tested the same way as LN1 but with half as many replicates. With LN2, gagL amplicons were confirmed in 1/21 and in 4/21 PCR reactions with USB and NEB master mixes, respectively. Water controls were always negative (total of 3 water controls for each 21 LN2-containing wells.

New LNCaP cells were purchased from ATCC and cultured. gDNA was prepared and designated LN3. LN3 was used in the same experiment carried out on LN2. No gagL PCR products were detected in any of the 48 reactions tested.

### *gag* PCR of DNA from PBMCs of control subjects in Ithaca, New York

In order to increase the number of control subjects in the study, we recruited 12 volunteers from Ithaca, New York, where a CFS/ME outbreak similar to the one that occurred in the Western New York area surrounding Lyndonville has not been reported. We observed a gagL PCR fragment only one time in an Ithaca control sample; as the result was not reproducible in subsequent assays, the sample was scored as negative.

### Absence of MLV-like *gag* sequences in gDNA samples from the New York City area

While we were carrying out the experiments on the Western New York samples, a number of reports began appearing that indicated problems with environmental contamination with mouse DNA as well as the presence of mouse DNA in common laboratory reagents. We therefore obtained a second set of samples from the office of Susan Levine, M.D. in Manhattan, New York. Single-round gagL PCR was performed on gDNA, and all assays were negative. These assays were performed in a different room in the Cornell laboratory that had improved environmental isolation over the one used for the experiments performed on the initial set of samples from the Lyndonville office. Research with mice had never been performed in any of the rooms used in this study; however, murine cell cultures and mice have been in use on the same floor of the laboratory building.

### Negative results with primers to XMRV envelope (*env*) sequences

Our original intention when this project commenced in 2010 was to compare XMRV envelope sequences from a population in Western New York (Lyndonville area) to those obtained in the Lombardi et al. [1] study that was carried out in Nevada, in order to determine whether there might be geographic variation in virus sequence. However, using the PCR assays described by Danielson et al. [16] or Hong et al. [17], no envelope sequences corresponding to any type of gammaretrovirus were ever detected in any gDNA or cDNA preparations we analyzed, even in samples where MLV-like *gag* sequences were identified. While amplicons were sometimes present in gel lanes, upon sequencing, the fragments were found to be human DNA non-specific amplicons. PCRs to assay for *pol* (Table S1) were also negative.

### Negative results with PCRs of conserved regions of MLV-like *pol*, *env*, and long terminal repeat (LTR) regions

Because of the possibility that an unusual gammaretrovirus might be present that would not be detectable with previously published primers, we predicted additional sets of primers from an alignment of exogenous and endogenous MLVs along with MLV-like sequences reported in prostate cancer and CFS. This alignment, which was kindly provided by Brian Foley from the Los Alamos National Laboratory, consisted of 111 DNA sequences, including the Rauscher, Friend, Moloney, and Graffi MLVs as well as C75BL/6J genome endogenous MLV subgroups [18], and XMRV and MLV-like sequences [9] that were available from NCBI in September 2010.

These primers were designed to amplify regions downstream of the regions assayed by the *gag* primers described above. Only the murine gammaretroviruses would be expected to be amplifiable with the new set of primers. A total of 13 primer pairs were used in PCR for regions *pol*, *env* and LTR (Tables S1 and S2) on samples that were previously positive for *gag* from PBMC and whole blood gDNA. All of the PCRs were negative for the respective retroviral targets. Amplicons of similar sizes to those that we expected were sequenced and were invariably non-specific amplification of human DNA.

**Figure 1:**
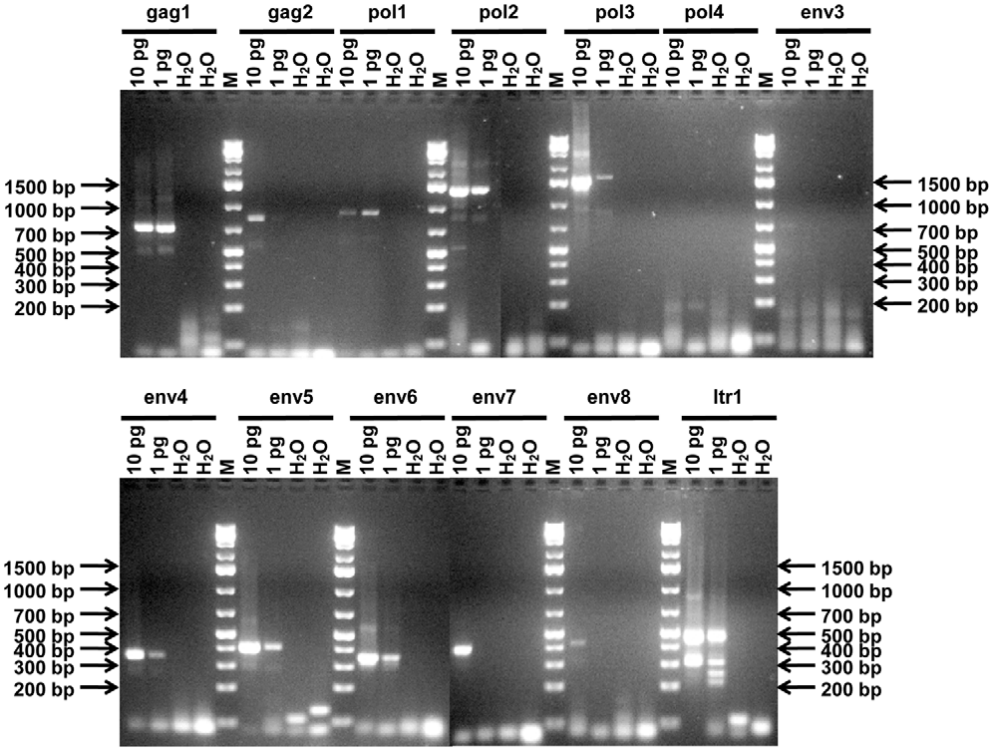
Amplification of conserved MLV-like regions from mouse tail DNA. 13 different PCRs (Table S2) were conducted with 10 pg or 1 pg mouse tail DNA as indicated. M – GeneRuler 1 kb Plus DNA Ladder, 75–20,000 bp (Fermentas) - selected fragment sizes are as indicated on the 2% agarose gel images. H_2_O – no template, PCR negative control.

Due to the multiple non-specific amplicon bands of varying sizes observed in these PCRs with human samples, we verified that the PCR primers were suitable for detection of gammaretroviral sequences with mouse tail DNA alone. The 13 different primers pairs resulted in the relevant products with mouse tail DNA (Fig. 1). The amount required for detection of viral sequences varied between 1 and 10 pg template DNA because the amplification of mouse endogenous retroviral sequences is dependent on the region and copy number of the target, which differs between mouse ERVs, LINEs and SINEs. All the amplicons were confirmed as of murine origin by sequencing and BLAST (NCBI), except for the env3 PCR reaction. Only a faint amplicon band was observed with 10 pg template DNA (Fig. 1). The primers for env3 PCR map to the more variable *env* region. We selected the most conserved regions for primer construction across the MLV and MLV-like genomes; however, the DNA we assayed was from one genotype of mouse only, C57BL/6J. Amplicons of two different sizes (between 300–400 bp and 400–500 bp) were obtained from mouse LTRs following ltr1 PCR. Endogenous MLVs are known to vary in size of LTRs.

### Sensitivity of PCR assays for *gag* and for mouse mtDNA and Intracisternal A Particle (IAP) DNA

**Figure 2:**
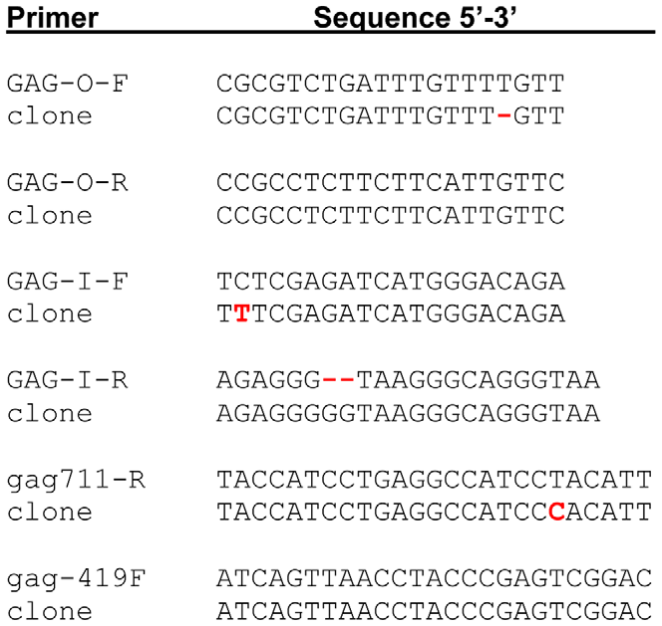
Alignment of primers with plasmid clone of 419F/1154R amplicon.

We determined the sensitivity of the gagO/gagI and gagL PCR assays by spiking 500 ng of human DNA with a plasmid carrying a cloned *gag* fragment obtained by amplifying a PBMC gDNA sample with the 419F/1154R primers. We were able to obtain *gag* amplicons with both assays in samples diluted so as to contain only a single copy of the plasmid. Thus both PCR assays are highly sensitive and can tolerate minor mismatches between the cloned target sequences and primers (Fig. 2).

**Figure 3:**
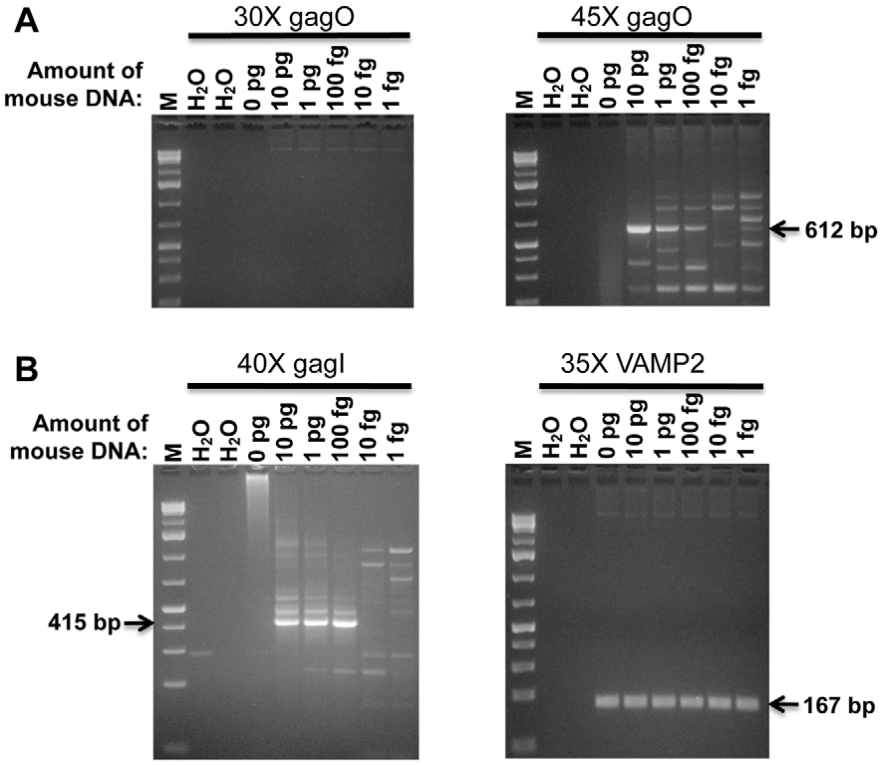
Detection sensitivity of gagO and gagI PCR with various amounts of mouse tail DNA in 500 ng of human whole blood DNA. (A) gagO amplicons were not visible after 30 cycles but with further cycling to a total of 45 cycles, gagO amplicons were amplified from 10 pg, 1 pg, and 100 fg mouse tail DNA. (B) 40 cycles of gagI PCR following 30 cycles of gagO PCR showed amplicons from 10 pg, 1 pg and 100 fg mouse tail DNA. As a positive control, VAMP2 PCRs resulted in amplicons from all samples with human DNA. M – GeneRuler 1 kb Plus DNA Ladder, 75–20,000 bp (Fermentas, Glen Burnie, Maryland). H_2_O – no template, PCR negative control.

In order to determine at what level we could detect mouse DNA if it were contaminating 500 ng of human DNA from blood samples, we performed experiments in which human whole blood DNA was spiked with 1 fg to 10 pg of mouse tail DNA (Fig. 3). While no amplicons were seen after 30 cycles of gagO PCR, we observed PCR products after an additional 15 cycles in samples spiked with 100 fg or more of mouse DNA. However, in our examination of human samples, we performed nested PCR of 30 cycles of gagO followed by 40 cycles of gagI, which resulted in greater amounts of amplicons than obtained by 45 cycles of single-round PCR with gagO (Fig. 3).

**Figure 4:**
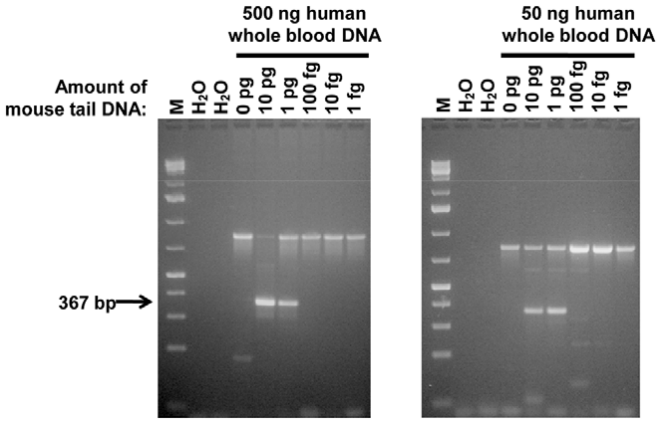
Detection sensitivity of gagL PCR with various amounts of mouse tail DNA. (A) 500 ng and (B) 50 ng of human whole blood DNA. gagL amplicons were detected in 1 pg of mouse tail DNA. M – GeneRuler 1 kb Plus DNA Ladder, 75–20,000 bp (Fermentas). H_2_O – no template, PCR negative control.

**Figure 5:**
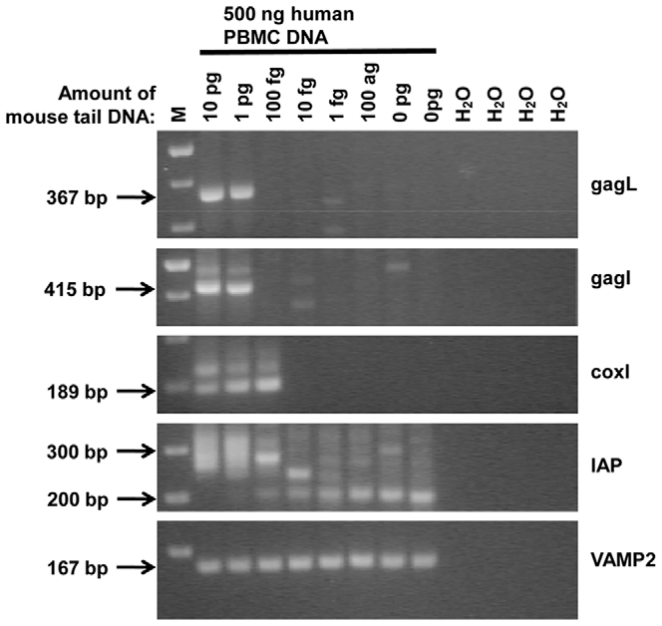
Sensitivity of PCR in detection of *gag* and mouse-specific targets in 500 ng of human PBMC DNA with a range of mouse tail DNA concentrations. The same master DNA dilution series was used as template in each of the PCR amplifications. gagL fragments were amplified from 1 pg mouse tail DNA, as were gagI fragments following gagO PCR. coxI following coxO PCR showed detection limit of 100 fg. IAP PCR was successful from 10 pg through 10 fg and variably sized fragments were observed between 200 and 300 bp; the lower amplicons of approximately 200 bp were sequenced and matched human DNA sequences. VAMP2 PCR was the positive quality control for human DNA. M – GeneRuler 1 kb Plus DNA Ladder, 75–20,000 bp (Fermentas). H_2_O – no template, PCR negative control.

We compared the sensitivity of the nested gagO/gagI PCR to the single-round gagL PCR and found that both PCR assays could detect 1 pg mouse tail DNA in 500 ng human gDNA (Fig. 4). The detection limit of 1 pg for the gagL PCR was identical whether 500 ng or 50 ng of human whole blood DNA was spiked with mouse DNA (Fig. 4). In order to determine whether mouse DNA contamination assays were more or less sensitive than our assays for *gag*, we spiked mouse DNA in 500 ng of human PBMC DNA and determined how much mouse DNA was needed before mtDNA and IAP assays could detect it. We were able to observe mtDNA sequences when only 100 fg was added to the human DNA (Fig. 5). The IAP assay was even more sensitive, as IAP amplicons were detected with 10 fg to 10 pg of mouse DNA added to human DNA (Fig. 5). With the IAP assay, variably sized fragments were observed between 200 and 300 bp. While a band present in the 10 pg lane was confirmed to be IAP by sequencing, the smaller amplicons of approximately 200 bp matched human DNA sequences.

**Figure 6:**
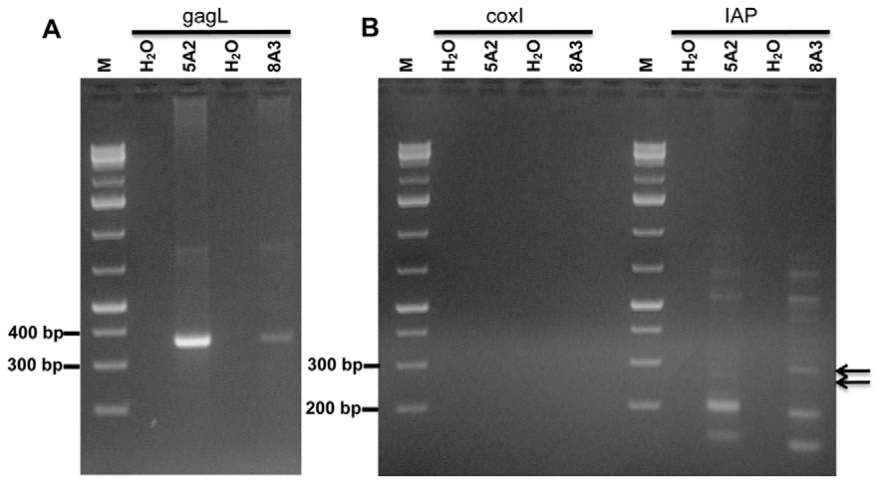
PCR detection of *gag* but not mouse sequences in 500 ng of whole blood gDNA. Fragments were separated on 2% agarose. (A) gagL fragments at 367 bp observed in 2 samples. (B) Neither mCOX2 nor IAP were amplified from the same 2 DNA samples, by nested coxO followed by coxI PCR, or IAP PCR, respectively. The amplicons of IAP PCR as indicated by the arrows were sequenced and were non-specific human DNA amplification, not of mouse origin. M – GeneRuler 1 kb Plus DNA Ladder, 75–20,000 bp (Fermentas). H_2_O – no template, PCR negative control. There is an empty lane between coxI and IAP lanes.

An example of amplification of *gag* sequences from two whole blood DNA samples, which were negative for mouse mtDNA and IAP, is shown in Fig. 6. Absence of mouse DNA cannot, however, rule out the possibility of environmental amplicon contamination.

### Analysis of *gag* sequences

All PCR products of the approximate size expected for *gag* sequences were sequenced. Occasionally, we had non-specific amplification in which the fragments matched human DNA according to analysis by BLAST (NCBI). We never used a positive control when assaying subject samples by PCR to prevent cross-contamination. Nevertheless, we are confident that negative results are not due to PCR inhibition because we regularly observed non-specific amplified fragments of variable sizes. All such fragments that were sequenced were amplified human regions.

**Figure 7:**
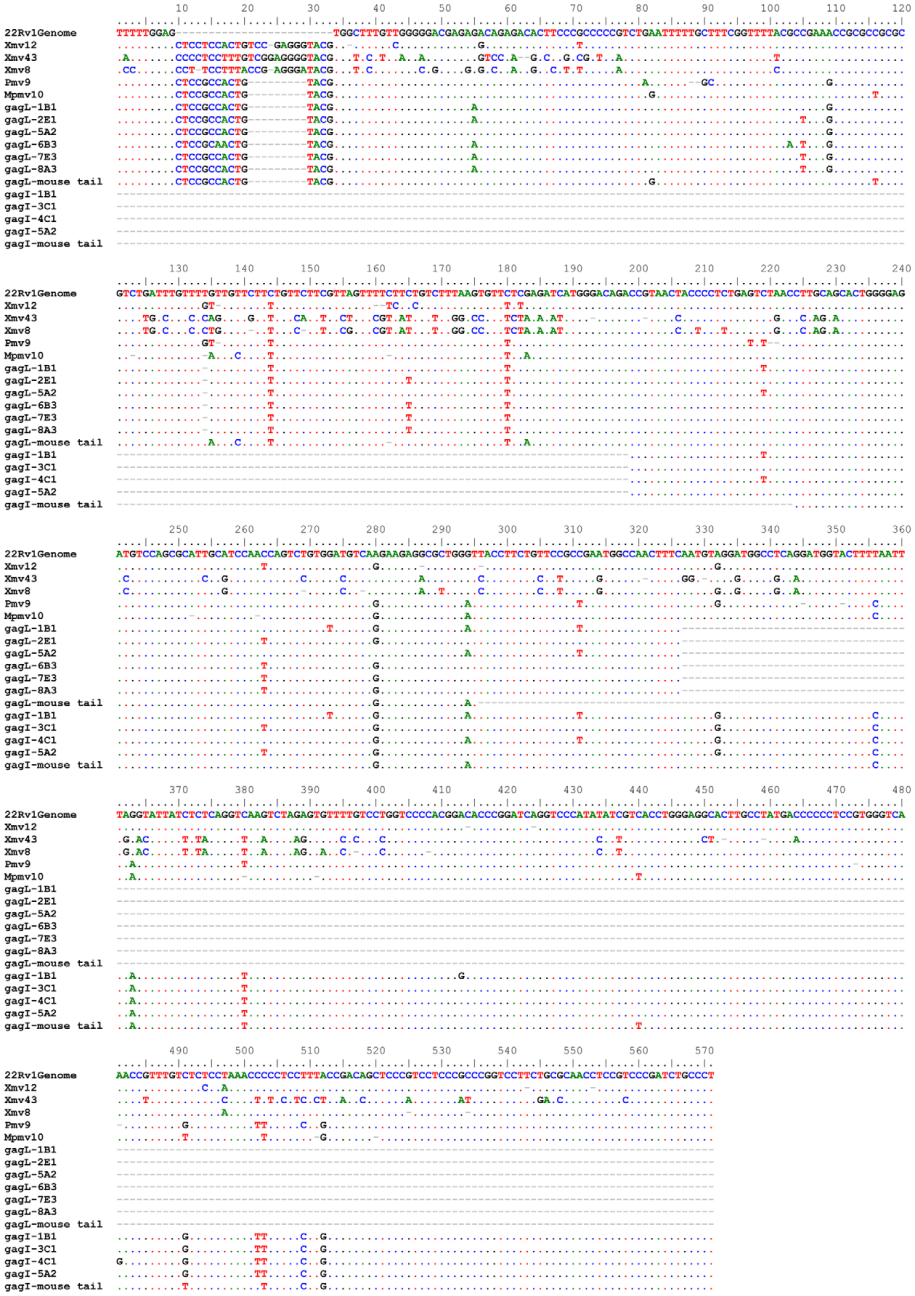
DNA sequence alignment of *gag* sequences with selected MLV-like sequences and mouse tail DNA sequences. Nucleotides that are identical to the reference 22Rv1Genome (Genbank FN692043) sequence are shown as dots and gaps are shown as dashes. Pmv9, Mpmv10, Xmv12, Xmv43, Xmv8 are endogenous polytropic, modified polytropic, and xenotropic viral sequences from the C57BL/6J genome that group into different clades according to Jern et al [18]. All other sequences shown were identified in this study. Sequences of nested gagO/gagI and gagL amplicons were distinct from representative MLV-like sequences, especially from the xenotropic sequences. Sequences amplified from blood and from mouse tail DNA were not 100% identical.

Of all the sequences we obtained (Fig. 7), only gagL sequence from 5A2 exhibited 100% identity with mouse [GenBank: AC153954.3; *Mus musculus* 10 BAC RP24-236E2 (Roswell Park Cancer Institute (C57BL/6J Male) Mouse BAC Library) complete sequence]. Mice of this genotype are used by a different laboratory located in our building. If the gagL product amplified was from mouse DNA contamination, we would expect to amplify mCOX2 and/or IAP from the same sample but this was not the case (Fig. 6). Further confounding is that the overlapping sequences between gagL and gagI sequences from 5A2 were not identical (Fig. 6). One explanation is that the gagI PCR of 5A2 resulted from amplicon or cross-contamination with gagI from 3C1, since the 2 amplifications yielded identical gagI sequences that were from nested gagO/gagI PCR. We cannot explain the gagL sequence from 5A2 as amplicon contamination since we did not find another sequence with 100% sequence identity. However, it is possible that the gagL sequence obtained was a hybrid sequence due to amplification from more than one template sequence, whether real or amplicon.

An alignment of the sequences we obtained with selected published MLV-related sequences is shown in Fig. 7 and Fig. S2. The differences between sequences amplified from mouse tail DNA and the samples from this study are apparent. The regions of sequence overlap of gagL and gagI were identical in 1B1 and 11F2-P4 but not in 5A2. gagL sequences were identical in the following samples: 2E1, 7E3, 8A3, 2F1-P6, 4F1-P6, 9F1-P6, 10F3-P6, 11F2-P4, and 12F2-P6. gagI sequences were identical in 3C1, 5A2, 11F2-P4, and 14F2-P4.

**Figure 8:**
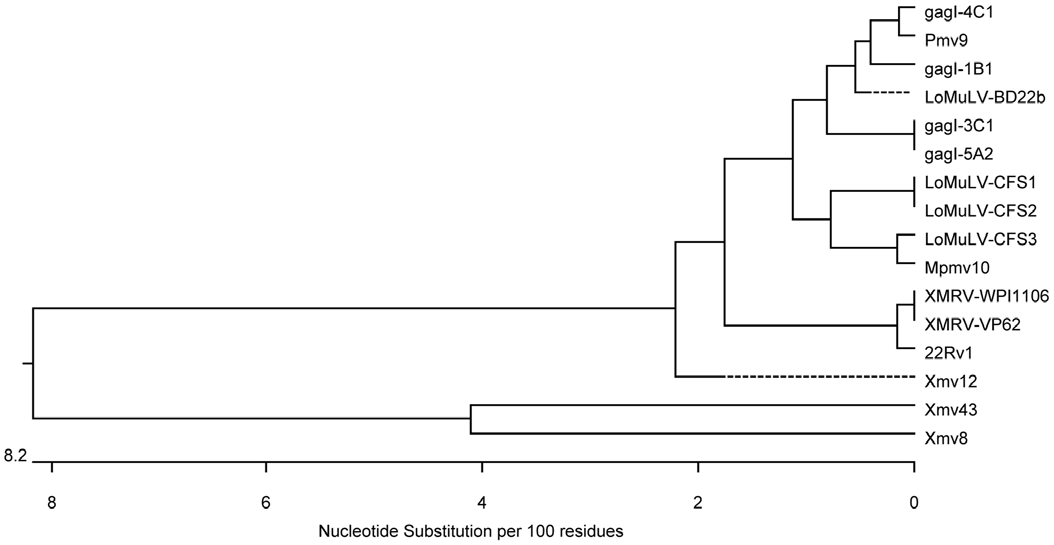
Phylogenetic tree created from alignments of sequences obtained from gagO/gagI PCR. Phylogenetic tree was created with DNASTAR MegAlign Version 8.1.2. by ClustalW (weighted) method. The LoMuLV sequences [9], XMRV-VP62 [19], and XMRV-WPI1106 [1] sequences were taken from Genbank. All other sequences shown were identified in this study.

**Figure 9:**
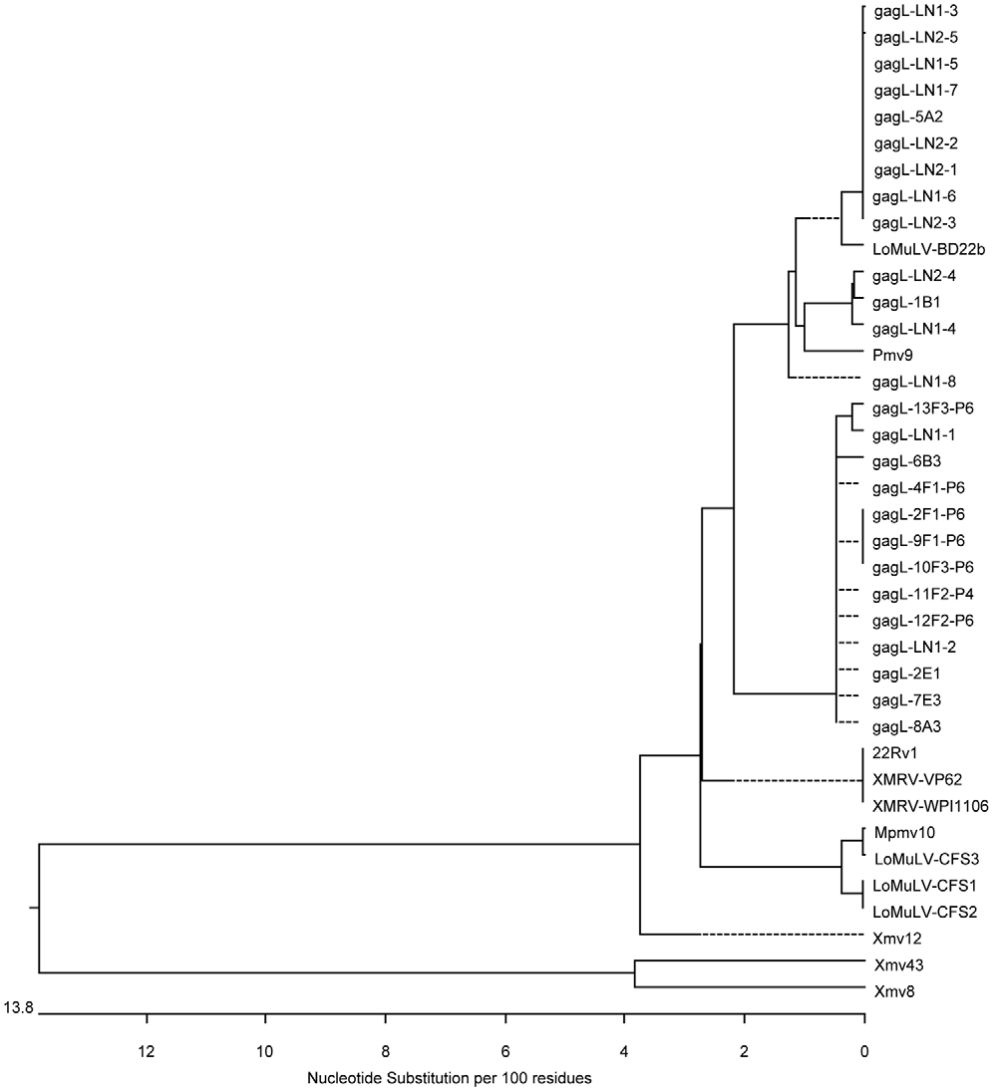
Phylogenetic tree created from alignments of sequences obtained from gagL PCR. Phylogenetic tree was created with DNASTAR MegAlign Version 8.1.2. by ClustalW (weighted) method. Sequences described in legends to Figure 7 and 8.

We have constructed phylogenetic trees with the gagI (Fig. 8) and the gagL sequences (Fig. 9). The trees include selected XMRV and MLV sequences [1], [18], [19] and MLV-like sequences obtained by Lo et al. [9]. The trees illustrate that the sequences amplified from the samples of this study were related to polytropic MLV-like sequences, and that all gagL sequences were distinct from 22Rv1 XMRV, which contains deletions in the *gag* leader region. There are differences among the sequences we obtained and between our sequences and those of Lo et al. [9]; however, it should be noted that relatively small sequences, approximately 400 nt, are being compared.

## Discussion

Like a number of other investigators, we have identified MLV-like *gag* sequences following PCR of nucleic acid samples from whole blood or PBMCs from human subjects. Nested PCR analysis of our initial batch of 30 samples resulted in a significant difference in frequency of *gag* PCR products between patients and controls; however, continued analysis failed to maintain this association. If sporadic contamination of reagents and/or environmental contamination was the source of the *gag* sequences, then a possible explanation for the initial association could be due to non-random receipt of patient and control samples. Although we were blinded to subject health status, we did not receive equal numbers of patient and control samples on the same day. If, for example, mouse DNA or MLVs were present on a day that 5 patient and 1 control samples were received, but absent on a day when 5 controls and 1 patient sample were received, then a higher frequency of patient versus control samples would appear positive for MLV-like DNA fragments. However, we did not observe any clear correlation between day of receipt of samples and whether they were positive by PCR for MLV-like sequences. While it is impossible to know whether or not contamination has occurred in another laboratory, one possible explanation for the much higher frequencies of positive patients versus controls in both the Lombardi et al. [1] and Lo et al. [9] studies could be the existence of reagent or environmental contamination at the time of assay of patient samples, but not when controls were assayed. In both of those studies, large batches of patient and control samples were assayed at separate times.

We attempted to replicate Lombardi et al.'s [1] finding of expansion of the virus signals following incubation of plasma with LNCaP cells; however, we found a curious lack of reproducibility of the positive PCR assays. It seems likely that our LNCaP cell line, which was maintained for many months, became contaminated at very low levels, or there was environmental contamination at the time of DNA preparation. We have no evidence for contamination of our enzymes or water. The enzymes we have used for our PCR experiments are not among those reported by others to be contaminated with mouse DNA and a large number of water controls were performed and found to be negative.

We have taken precautions against environmental contamination, including use of UV-irradiated work spaces and tubes and isolation of the laboratory room from individuals who work with mice or mouse cells. All of our mouse tail DNA spiking experiments were performed in a separate laboratory. One possible source of contamination is PCR product carryover from prior experiments. While one group used the dUTP-UDG method to prevent carryover of PCR products [20], our preliminary experiments suggested that this technique reduces the sensitivity of our PCR assays, which is consistent with a subsequent report [21]. We chose instead to reduce the possible environmental contamination of PCR assays by developing a single-round assay that can detect spiked plasmid DNA with the same sensitivity as the previously described gagO/gagI nested PCR. When this assay, which requires less manipulation than nested PCR, was used on a new set of DNA samples, we did not detect MLV-like *gag* sequences.

Our data indicate that the IAP assay [10] with the specified primers and conditions is extremely sensitive and can detect the presence of mouse DNA at lower levels than the mitochondrial DNA assay when the source of deliberately introduced mouse cellular DNA is from mouse tail. One issue with mtDNA assays for the presence of mouse DNA is that the tissue source of the contamination will affect how readily the presence of a mouse cell can be detected. Liver, brain, and heart cells contain far more mitochondrial DNA than spleen or bone marrow cells, for example [22]. In contrast, the amount of IAP DNA per cell of a particular mouse species will be constant, though different species could vary somewhat in IAP content. As long as the IAP assay is negative, a positive *gag* signal with the nested gagO/gagI PCR must not be due to the presence of mouse DNA. However, the IAP assay does not allow determination of contamination due to the presence of *gag* RNA, nor can it reveal the presence of PCR fragment carryover between experiments.

While it now appears likely that the virus named XMRV originated as a result of passage of cell lines through mice [23], the inquiry into the possible connection of this virus to prostate cancer and CFS/ME has generated some important information. Before the attempts to replicate the Lombardi et al. [1], it was not known that a number of cell lines were infected with XMRV or related MLVs, possibly causing misinterpretation of data when such lines were used in various experimental protocols. How readily this type of virus can spread from one cell line to other cell lines was also not appreciated [24]. Furthermore, the frequent contamination of reagents [12], [25]–[27] and lab environments with mouse DNA [10], [11], [28], despite standard precautions, has been unexpectedly high. The necessity for screening for the presence of mouse DNA has led to the development of a useful assay [10]. Should there be a future zoonotic transmission of MLV or other mouse viruses into the human population, the scientific community will be better prepared to verify its presence.

Whether there are unknown retroviruses that are inciting factors in CFS/ME remains unknown. The PCR primers that we and others have employed for screening for XMRV and MLV-like sequences will allow detection of only a subset of viruses related to MLV. These PCR assays would not have amplified sequences from common feline leukemia viruses or gibbon ape leukemia viruses, even though they also are in the gammaretrovirus family. In fact, Elfaitouri et al [29] have pointed out that most of the primer sets that have been used to study CFS/ME samples would not even detect all groups of MLVs. Had it not been for the 2009 report [1] associating XMRV with CFS/ME, we would not have chosen PCR amplification for identification of viruses in this population. Less specific methods such as virus microarrays or high-throughput DNA sequencing are more suitable for detection of unknown agents that may be associated with disease states. Their application should be fruitful in identification of pathogens that may more frequently infect CFS/ME patients, either as a cause or consequence of the illness, and will be instrumental in verifying whether or not gammaretrovirus infections exist in humans and/or whether or not an unknown viral infection is associated with CFS/ME.

## Supporting Information

Table S1 **PCR primers used in this study.**
(XLS)

Table S2 **PCR conditions used in this study.**
(XLS)

Figure S1 **DERSE cells expressing green fluorescent protein following incubation with virus from 22Rv1.** Image was acquired with a Zeiss 710 confocal microscope.
(TIF)

Figure S2 **Alignments to XMRV and MLVs of all sequences obtained by PCR in this study.** Mpmv and pmv are from reference 22Rv1 sequence: Genbank FN692043. Polytrop 15: FJ544577. Polytrop 51: FJ544578.
(TIF)
