# Supplementary figures and images for: Sensitivity of PCR Assays for Murine Gammaretroviruses and Mouse Contamination in Human Blood Samples
Source: PLoS One. 2012 May 21;7(5):e37482. doi: 10.1371/journal.pone.0037482 (PMC3357399; doi:10.1371/journal.pone.0037482)

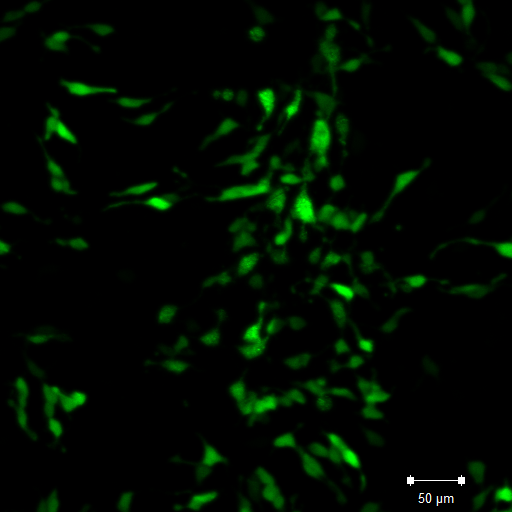

Supplement: Figure S1 — DERSE cells expressing green fluorescent protein following incubation with virus from 22Rv1. Image was acquired with a Zeiss 710 confocal microscope. (TIF) [file pone.0037482.s003.tif]

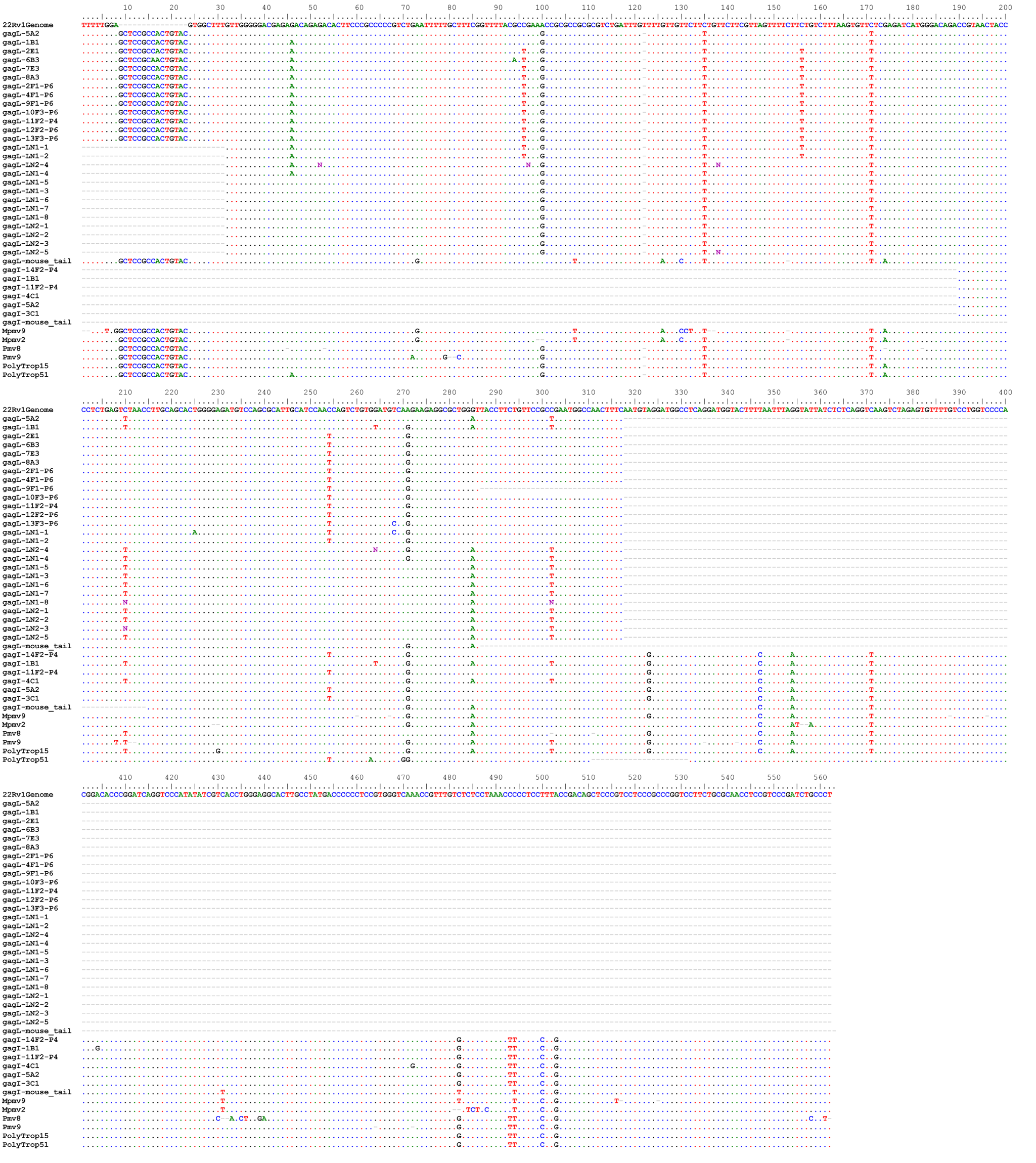

Supplement: Figure S2 — Alignments to XMRV and MLVs of all sequences obtained by PCR in this study. Mpmv and pmv are from reference 22Rv1 sequence: Genbank FN692043. Polytrop 15: FJ544577. Polytrop 51: FJ544578. (TIF) [file pone.0037482.s004.tif]
